# Deep Temporal Convolution Network for Time Series Classification

**DOI:** 10.3390/s21020603

**Published:** 2021-01-16

**Authors:** Bee Hock David Koh, Chin Leng Peter Lim, Hasnae Rahimi, Wai Lok Woo, Bin Gao

**Affiliations:** 1 School of Engineering, Nanyang Polytechnic, Singapore 569830, Singapore; peter_lim@nyp.edu.sg; 2 Department of Computer and Information Sciences, Northumbria University, Newcastle upon Tyne NE1 8ST, UK; hasnae.rahimi@northumbria.ac.uk (H.R.); wailok.woo@northumbria.ac.uk (W.L.W.); 3 School of Automation Engineering, University of Electronic Science and Technology of China, Chengdu 611731, China; bin_gao@uestc.edu.cn

**Keywords:** sensor signals, neural networks, time series classification

## Abstract

A neural network that matches with a complex data function is likely to boost the classification performance as it is able to learn the useful aspect of the highly varying data. In this work, the temporal context of the time series data is chosen as the useful aspect of the data that is passed through the network for learning. By exploiting the compositional locality of the time series data at each level of the network, shift-invariant features can be extracted layer by layer at different time scales. The temporal context is made available to the deeper layers of the network by a set of data processing operations based on the concatenation operation. A matching learning algorithm for the revised network is described in this paper. It uses gradient routing in the backpropagation path. The framework as proposed in this work attains better generalization without overfitting the network to the data, as the weights can be pretrained appropriately. It can be used end-to-end with multivariate time series data in their raw form, without the need for manual feature crafting or data transformation. Data experiments with electroencephalogram signals and human activity signals show that with the right amount of concatenation in the deeper layers of the proposed network, it can improve the performance in signal classification.

## 1. Introduction

With the proliferation of sensors, time series data are now widely available. They are encountered in many real-world applications, such as human activity recognition [1], identification of epileptic condition [2], diagnostic of heart diseases [3], defect detection [4], and many others [5,6].

Due to the nonstationary, nonlinear, and noisy nature of real-world time series data, it is daunting for the human cognitive process to classify the signals. This is, however, not a problem for machine learning, and many methods have been devised by researchers to solve the problem [7]. They can generally be categorized as feature-based, distance-based, and neural network-based.

The traditional approach in machine learning to classify time series data is feature-based. It models the time series as a generative process [8] by assuming a certain time series model, such as the autoregressive model [9], the linear dynamic system [10], and the hidden Markov model (HMM) [11]. After estimating the model parameters from the data, they are then used as features in a machine learning classifier [12,13].

That approach needs domain knowledge, which is often unavailable. A more practical approach is the discriminative approach. It is based on the distance between two time series. To classify a data instance, the distance of the data instance from those in the training set will be computed, which is then used in the k-nearest neighbor classifier [14]. The default distance measure is the Euclidean distance. There are many alternative elastic distance measures in the literature that can give better results, the most common being dynamic time warping (DTW) [15], longest common subsequence (LCS) [16], edit distance with real penalty (ERP), and edit distance on real sequence (EDR) [17]. They are able to shrink or stretch the time axis to find the best alignment between the time series and obtain the smallest distance between them [18]. The performance of distance-based methods can be boosted by combining multiple classifiers with different elastic measures in a single ensemble [19]. One such ensemble is Collective of Transformation-based Ensembles (COTE). It makes use of 35 classifiers over different distance measures in the time and frequency domains [20]. It is, however, computationally intensive to cross-validate the hyperparameters of so many classifiers and elastic measures [21].

The neural network-based method, also known as deep learning, is an end-to-end method. It is exciting, as it can extract features from the raw signals without the need to perform feature engineering or specify the distance measure [22,23].

There are many network architectures for deep learning. Most of them are for image classification [24], and only some are for the one-dimensional multivariate time series data [25]. A one-dimensional time series is a function of a single independent variable, usually time. When there is more than one channel of such signal, such as the channels of an electrocardiogram, it forms a one-dimensional multivariate time series.

The baseline network for time series classification is the multilayer perceptron (MLP) [26], which consists of hidden layers that are fully connected. Other networks that have been proposed for the time series classification include the fully convolutional networks (FCN) [27], multichannel deep convolution neural network (MC-DCNN) [28], residual network (ResNet) [29], and the echo state network (ESN) [30]. They make use of layers such as the batch normalization (BN) layer [31] and the global average pooling (GAP) layer, as well as shortcut links [32] between layers, to stabilize the learning process to reduce the vanishing gradient effect. Besides time series classification, deep neural networks such as pyramid recurrent neural network [33] can be used for change point detection to detect abrupt or gradual changes in the signal characteristics, achieved by transforming the time series data into a pyramid of multiscale feature maps in a trainable wavelet layer. In addition, an ensemble of neural networks can be used to boost the performance of time series classification [34,35,36]. InceptionTime [37] is one where a set of five different models formed by cascading multiple deep convolution neural networks, called the Inception module [38], are used.

The foundation of all these networks is composition. In composition, the output of a layer becomes the input of the next layer. This kind of compositional structure matches with the compositional function of many natural signals such as image, text and speech [39]. These signals have what is called the property of locality [40], which means that the features formed by neighboring points are related to one another at different scales and time. In this work, we build upon the above idea and propose a new deep learning network to exploit the compositional locality of the time series data at all levels of the network, including the deeper layers of the network. The aim is to match the network with the complex data function of a highly varying time series, so that shift-invariant features can be extracted layer by layer at different time scales, and thus boost the classification performance. The proposed network makes two key contributions to the neural network architecture, which are (1) the use of data processing and the concatenation operation to introduce the temporal context to the deeper layers, and (2) a matching learning algorithm for the revised network, based on the idea of gradient routing in the backpropagation path.

The remainder of this paper is organized as follows: Section 2 shows the architecture of the proposed network and explains how the temporal context can be represented, distributed, and learnt in many layers. Section 3 describes the proposed methodology to concatenate the temporal context, prepare the data, and learn by backpropagation with gradient routing. Section 4 describes the data experiment on a multichannel electroencephalogram data set and a human activity recognition data set, with the results and some discussion. Section 5 concludes the paper.

## 2. Network with Temporal Context

**Figure 1:**
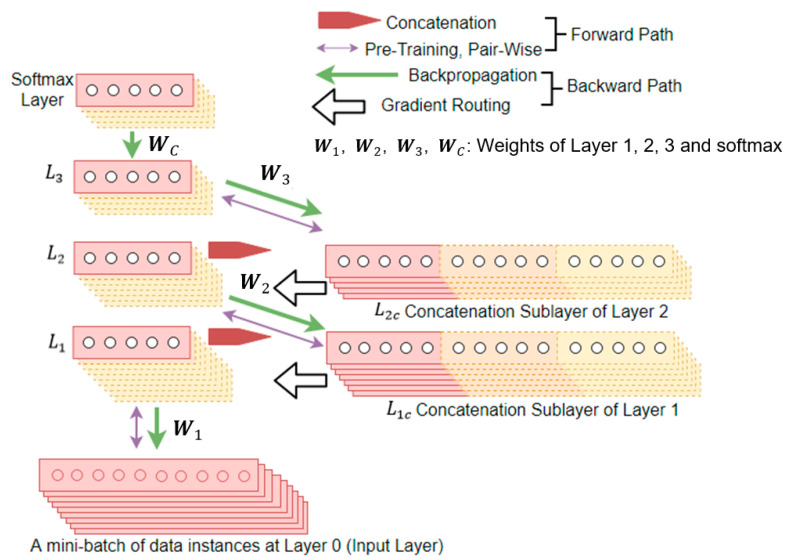
Architecture of the proposed deep temporal convolution network, shown with three hidden layers and a final classifier.

Figure 1 shows the architecture of the proposed network. Starting from the bottom of the figure, the time series data are first arranged in the time delay representation in mini-batches, with each mini-batch consisting of a small number of data instances, for example 8 or 32 [41]. The output of each of the hidden layers is rearranged by the concatenation operation, resulting in a new input for the next hidden layer. The network weights are located between the new input and the next hidden layer. These weights are trained by pretraining [42] in the forward path, and then by backpropagation [43] with gradient routing in the backward path.

The proposed network addresses the following problems: (A) representation of temporal context, (B) distribution of temporal context, and (C) learning with many layers. They are explained in the following subsections.

### 2.1. Representation of Temporal Context

For a signal to be classified by a neural network, it will have to be represented in what is called the time delay representation [44]. This can be done easily for discrete time series x with N time series elements, i.e., sample points, at constant sampling rates, $x=\left(x_{1},\ldots ,x_{N}\right)$. Simply slide a window of fixed length w across the signal with stride s, s<w. The result is a set of overlapping segments. Each segment is a data vector containing w samples.

**Figure 2:**
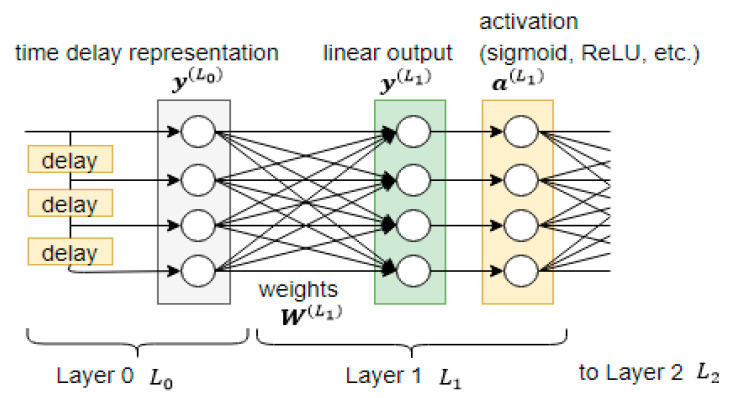
A tapped delay line at the input of a time-delay neural network (TDNN), w=4, s=1.

The data vector, used at the input of the neural network, can be viewed as a tapped delay line used for convolution, as shown in Figure 2. A neural network that treats its input in this way is called the time-delay neural network (TDNN). It was introduced by Waibel et al. [45] and has been used in many time series applications, such as human sound location [46] and the detection of Parkinson disease [47].

The sample points in the data vector are the lag observations of the signal. They contain the time-dependent patterns that the algorithm can learn. The amount of overlap between any two neighboring segments is shown in Equation (1) below.

(1) $$overlap\;\%=\frac{w-s}{w}\times 100\%$$

The overlapping of the segments is important. It ensures that the nonstationary features are represented at different time positions. This makes the training of a shift-invariant model possible, so that there is no need to provide the exact starting and ending points of the temporal features.

The sliding window method, used at the input to create the time delay representation, is sufficient for good performance in time series classification. The problem with this approach is the loss of temporal context in the hidden layers, and so the features learned in the hidden layers are no longer time-invariant.

### 2.2. Distribution of Temporal Context

**Figure 3:**
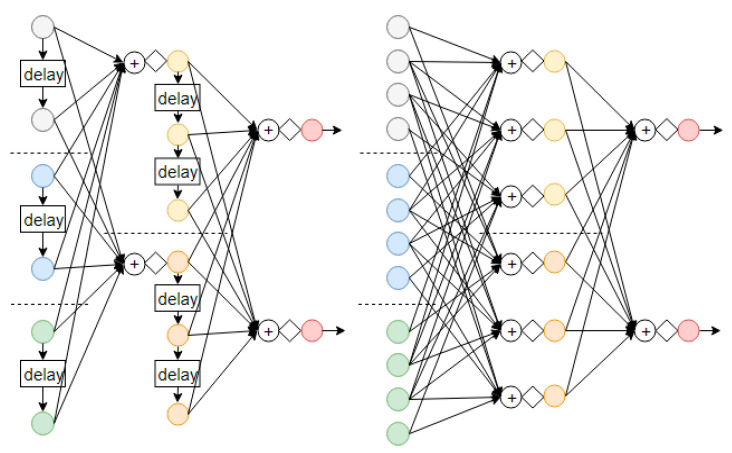
A distributed TDNN (left) and its equivalent network (right). The empty circle represents a sample in the data vector. The circle with a plus sign represents the summation operation. The diamond represents a nonlinear activation function such as the sigmoid function.

In Figure 2 shown earlier on, the data vector at the input of the neural network was a tapped delay line. To distribute the temporal context to the hidden layers, the data at the hidden layers can likewise be stored as tapped delay lines. This is shown in Figure 3. The left-hand side shows the distributed TDNN with an input layer of 3 units (each unit with a 2-tap delay line), a hidden layer with 2 units (each unit with a 3-tap delay line), and a final output layer with 2 units. The right-hand side shows the equivalent network. It is a plain neural network which is static with no tapped delay line.

By comparing the distributed TDNN on the left-hand side and the equivalent network on the right-hand side, it is found that the number of unique weights for both of them are the same. This is despite the equivalent network having more nodes than the distributed TDNN. The reason for this is that the nodes in the equivalent network are not fully connected. For those nodes that are connected, many of them share the weights by simply reusing the weights by shifting them down.

The idea of weight sharing is used in the proposed deep temporal convolution network in the form of concatenation. The concatenation operation can be repeated in a deep network, which is not amendable to the distributed TDNN in Figure 3 due to the computational issue of exploding and/or diminishing gradient when the number of hidden layers is increased [48].

### 2.3. Learning with Many Layers

To overcome the computation problem, it is necessary to initialize the network weights to some “good” values [49]. This is possible with pretraining as is used in the Deep Belief Net—Deep Neural Network (DBN-DNN) [42].

The DBN-DNN is a static network that comprises two parts: a stack of restricted Boltzmann machines (RBMs) [50], collectively known as the DBN, and a final output classier (for example, a softmax layer) on top of it.

**Figure 4:**
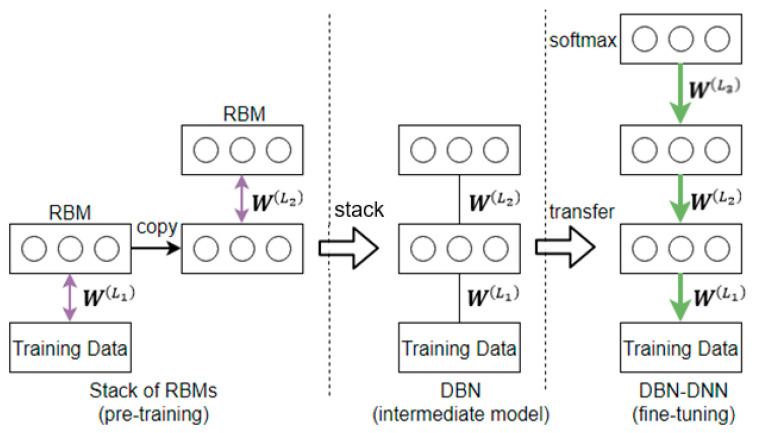
The training process of a DBN-DNN, consisting of pretraining, the intermediate model, and fine-tuning.

The training process of the DBN-DNN is divided into two stages, comprising the pretraining stage and the fine-tuning stage. This is shown in Figure 4 below.

From Figure 4 above, it can be seen that the pretraining stage applies only to the DBN, which is the intermediate model of the DBN-DNN. It does not involve the softmax layer or the target labels. It is thus an unsupervised training process. This is in contrast with the fine-tuning stage, which is a supervised training process.

The pretraining is pair-wise and operates in the forward direction [51]. It starts at the bottom of the DBN, where a pair of layers, nominally the visible layer and the hidden layer, forms the RBM. The process of unsupervised training by contrastive divergence [52] is run on the RBM. Upon convergence, the weights between the two layers will become fixed, and the same process of unsupervised training by contrastive divergence will then be brought forward to the next pair of layers. In moving forward, the output (hidden layer) of the previous RBM will become the input (visible layer) of the current RBM.

After pretraining, the weights in the DBN are transferred to the DBN-DNN, where together with the weights of the softmax layer, they are fine-tuned by backpropagation.

A DBN-DNN trained in this manner (pretraining in the forward path, followed by fine-tuning in the backward path) will make the network relatively immune to overfitting.

The limitation of the DBN-DNN is that the temporal context is not distributed to the deeper layers of the network. To do so, we propose using data processing based on the concatenation operation within the DBN-DNN. We will provide the matching learning algorithm for the revised network.

## 3. Proposed Methodology

In this section, we explain the concept of concatenating temporal context in the deeper layers, the details of preparing the data to maintain short-term temporal order in the mini-batches, and the backpropagation with gradient routing method for the learning process.

### 3.1. Concatenate the Temporal Context

In this work, the temporal context of the time series data is chosen as the useful aspect of the data that is passed through the network [53]. The temporal context consists of neighbors that are next to each other in time.

**Figure 5:**
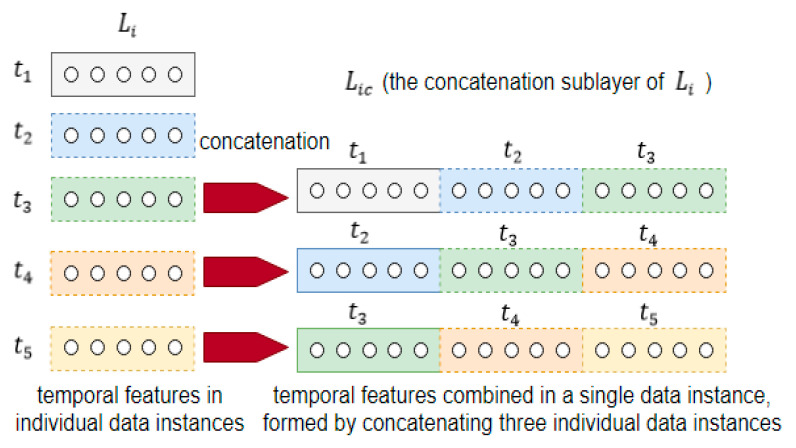
An illustration of the formation of the concatenation sublayer at TS=3.

An example of the concatenation operation is shown in Figure 5 below. The figure shows 5 data instances in the layer $L_{i}$ at time $t_{1}$, $t_{2}$, $t_{3}$, $t_{4}$, and $t_{5}$. They are combined to become the new data instances in the layer $L_{ic}$, which is the concatenation sublayer of the input in $L_{i}$.

The combination of the data instances is according to their natural time order. It must not be random. For example, in Figure 5 above, the data instances at $t_{1}$, $t_{2}$, and $t_{3}$ form a new data instance, while the data instances at $t_{2}$, $t_{3}$ and $t_{4}$ form another new data instance. As such, the new data instances in $L_{ic}$, formed by the concatenation operation, will have more temporal context than the individual data instances in $L_{i}$. They will act as the new input for the next hidden layer $L_{i+1}$.

In this work, the amount of concatenation will be described by a variable known as the time steps, TS. It is a hyperparameter of the proposed network. In the example in Figure 5 above, the value of TS is 3. This is because each concatenation consists of 3 data instances.

The data instances in the concatenation sublayer are all obtained with the same set of weights before they are concatenated. Concatenation can therefore be viewed as weight-sharing.

The 5 individual data instances in Figure 5 above form what is known as a mini-batch, a term used to differentiate from the term “batch” as used in “batch gradient descend” where it refers to the entire data set. All operations in the proposed network, including data preparation and network learning, will be done in mini-batches rather than by individual data instances.

### 3.2. Preparing the Data

The time series data and their labels will have to be reformatted so that the shift-invariant temporal context can be learnt. This reformatting includes the following processes:

(1) Maintain short-term temporal order within a mini-batch
(2) Create mini-batches that overlap with their neighbors
(3) Pool the count of the target labels through the deeper layers

The first two steps are used to prepare the time series data for use as the input of the network. The third step is used to associate the training data to the correct target labels for learning.

#### 3.2.1. Short-Term Temporal Order

To have short-term temporal order, the data instances in the mini-batches must be kept in their natural time order. The mini-batches will then be shuffled to shatter the long-term time order.

Maintaining short-term temporal order clears up the following dilemma faced by the proposed network.

On one hand, the concatenation of the data instances is only meaningful if the data instances are in their natural time order, otherwise randomness will be injected into the concatenated data and worsen the network performance.

On the other hand, each of the data instances must be a sample that is independent and identically distributed, otherwise the simple output pattern in the time series data set will be learnt by the network. As this pattern is incidental to the training data and unlikely to recur in the test data, overfitting the network [54] to it will produce poor test result in spite of the good training result.

The use of short-term temporal order solves the aforementioned dilemma. In addition, it fits into the practice of using mini-batches for computational efficiency. The size of a mini-batch is typically a small number from 8 to 32. Keeping the mini-batch size to 32 or less provides improved generalization performance with a small memory footprint [41]. It should be 8 or more to cater for the need to form the concatenation sublayers in the proposed network.

#### 3.2.2. Mini-Batches That Overlap

The mini-batches should overlap with their neighbors so that the network can be shift-invariant and less dependent on the precise location of the temporal feature within the mini-batch. This is a necessary step and is in addition to the time delay representation. The mini-batches will have to be randomized before they are passed through the proposed network for training. This will ensure that the temporal context maintained by short-term temporal order in the mini-batch can be learnt in a shift-invariant manner.

**Figure 6:**
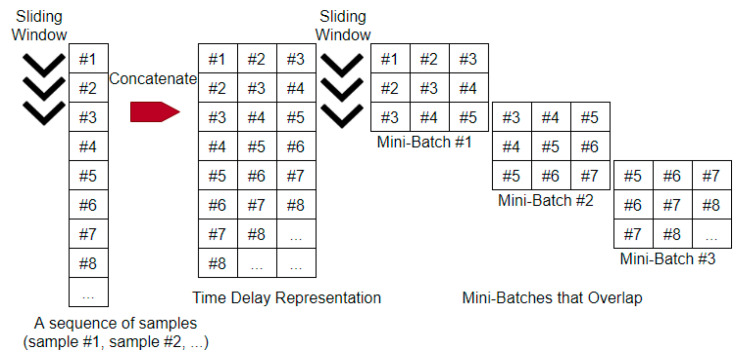
A two-stage sliding window to create mini-batches that overlap.

Figure 6 shows the proposed two-stage sliding window method to create mini-batches that overlap with their neighbors. On the time series, which is a sequence of samples in their time order, slide a fixed-size window along it to create the time delay representation. On the time delay representation thus created, slide another fixed-size window to create the mini-batches that overlap with each other.

Within each of the mini-batches, there is an unequal contribution of the data instances. For example, in the first mini-batch in Figure 6 above, the data instance #1 appears once, whereas the data instance #3 appears three times. The unequal contribution will be largely eradicated when all the overlapping mini-batches are considered as a whole. It will not affect the effective training of the network, as it is similar in nature to the random sampling of nonstationary time series.

#### 3.2.3. Pool Target Labels through the Deeper Layers

As there are many samples in a data vector, their target label in common has to be decided by majority voting. While this could be done at the input layer of the TDNN, it should instead be delayed until the final classifier in the proposed network. This is because the concatenation operation will add more data to the deeper layers, and so it is necessary to add the count of the target labels as and when the concatenation operation is done.

A simplistic scheme will distort the actual class distribution. For example, if there are three data vectors, two of them class 1 and one of them class 2, then class 1, being the majority class, will be deemed as the target label of the concatenated vector in this simplistic scheme. The distortion occurs because the target labels of the data vectors are themselves the result of majority voting in the previous layer and have lost some of the information due to the summarization.

**Figure 7:**
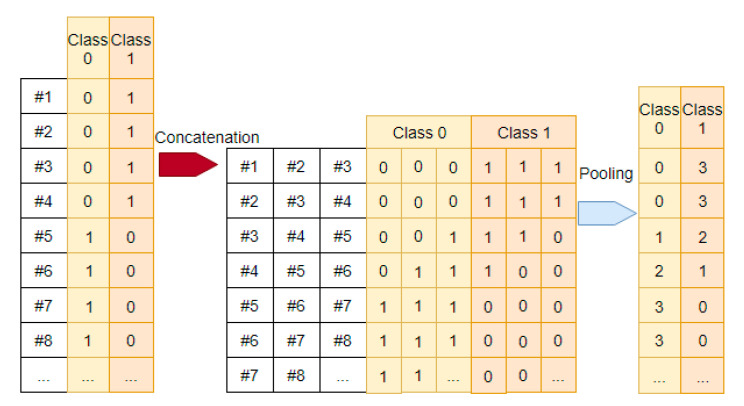
Pooling of the class counts of newly concatenated data. The diagram shows how the count of each class in a newly concatenated data vector is pooled from the count of the classes in the input of the concatenation.

The proposed solution is to pool the count of the target labels and accumulate them through the deeper layers. The target labels are first expressed in the one-hot encoding format so that there is one category per class. This allows the count of each class to be updated after the concatenation operation, as shown in Figure 7.

The updated counts of the classes at the last layer (the final classifier) are then used by the majority voting scheme to decide on the final target label of the data. In case of tie, a pseudorandom number generator can be used to decide on the class of the target label. Pooling the target labels through the deeper layers of the deep temporal convolution network avoids the loss of information, thus enhancing the validity of the target labels.

#### 3.2.4. Learn by Backpropagation with Gradient Routing

The proposed network can be trained in two stages: pretraining as a stack of RBMs and fine-tuning of the entire network by backpropagation with gradient routing.

##### Pretraining

The pretraining of the proposed network is by the same pair-wise unsupervised training using contrastive divergence as the DBN-DNN. The difference is that now, the visible layer of the RBM is the concatenation sublayer rather than the hidden layer.

**Figure 8:**
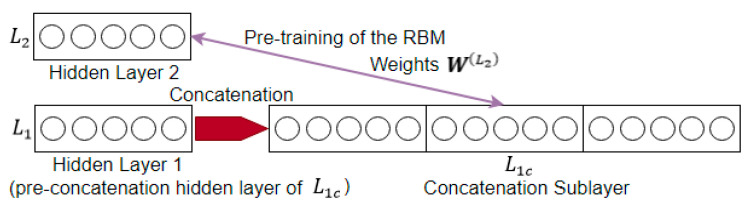
Restricted Boltzmann machines (RBM) in the deep temporal convolution network.

This is illustrated in Figure 8 below. In the DBN-DNN, the RBM would be between $L_{1}$ and $L_{2}$; but in the proposed network, the weights are located between the concatenated sublayer $L_{1c}$ and the next hidden layer $L_{2}$, and so the RBM is formed between $L_{1c}$ and $L_{2}$ instead.

##### Backpropagation with Gradient-Routing

In general, the weights in a network can be updated by gradient descend, as shown in Equation (2) below.

(2) $$W\leftarrow W-\alpha \frac{\partial J\left(W\right)}{\partial W}$$

In Equation (2), J(W) is the cost function. It will subsequently be abbreviated here as the error E. To update the weights of a layer in a network with many layers, say that of the i-th layer, the contribution of the i-th layer to the error E should be determined precisely. That contribution, sometimes referred to as the delta or the sensitivity, is denoted as $\delta^{\left(L_{i}\right)}$, where $L_{i}$ is the i-th layer. It is, by definition, the derivative of the cost function E with respect to the linear output $y^{\left(L_{i}\right)}$, and is shown in Equation (3) below.

(3) $$\delta^{\left(L_{i}\right)}≜\frac{\partial E}{\partial y^{\left(L_{i}\right)}}$$

The determination of $\delta^{\left(L_{i}\right)}$ should proceed layer by layer in the backward direction. There is one catch, though. All the sections in the backward path must be able to be linked together by the chain rule of derivative.

**Figure 9:**
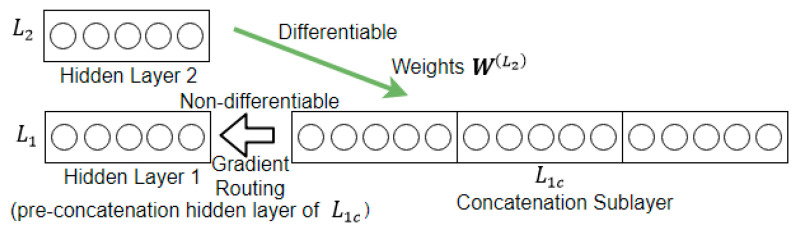
Backward path from concatenation sublayer to the preconcatenation hidden layer.

The above condition cannot be satisfied by the proposed network. This is because the concatenation operation is not a smooth function, and so the backward path from the concatenation sublayer to the preconcatenation hidden layer is not differentiable. In Figure 9 below, the nondifferentiable section is from $L_{1c}$ to $L_{1}$.

By the chain rule of derivative, the contribution of the i-th layer $\delta^{\left(L_{i}\right)}$ can be factorized as the product of four terms, as shown in Equation (4) below.

(4) $$\delta^{\left(L_{i}\right)}≜\frac{\partial E}{\partial y^{\left(L_{i}\right)}}=\frac{\partial E}{\partial y^{\left(L_{i+1}\right)}}\frac{\partial y^{\left(L_{i+1}\right)}}{\partial a^{\left(L_{ic}\right)}}\frac{\partial a^{\left(L_{ic}\right)}}{\partial a^{\left(L_{i}\right)}}\frac{\partial a^{\left(L_{i}\right)}}{\partial y^{\left(L_{i}\right)}}$$

By tracing through the four terms in Equation (4), it can be seen that the delta passes through the following parts of the network: 1. $y^{\left(L_{i+1}\right)}$, the linear output of the upper hidden layer $L_{i+1}$, 2. $a^{\left(L_{ic}\right)}$, the activation of the layer $L_{ic}$, which is a concatenation sublayer, 3. $a^{\left(L_{i}\right)}$, the activation of the layer $L_{i}$, which is the preconcatenation hidden layer, and 4. $y^{\left(L_{i}\right)}$, the linear output of the layer $L_{i}$.

The first two terms in Equation (4) pose no problem for computation. The first term is, by definition, the delta of the upper layer $\delta^{\left(L_{i+1}\right)}$, and so is available from the previous calculation during backpropagation. The second term is, by differentiation, the weight $W^{\left(L_{i+1}\right)}$ of the upper layer $L_{i+1}$, since $y^{\left(L_{i+1}\right)}=W^{\left(L_{i+1}\right)}a^{\left(L_{ic}\right)}$. With these two terms available, their product, denoted as $\frac{\partial E}{\partial a^{\left(L_{ic}\right)}}$ in Equation (5) below, can be computed directly by multiplication according to the chain rule.

(5) $$\frac{\partial E}{\partial a^{\left(L_{ic}\right)}}=\frac{\partial E}{\partial y^{\left(L_{i+1}\right)}}\frac{\partial y^{\left(L_{i+1}\right)}}{\partial a^{\left(L_{ic}\right)}}=W^{\left(L_{i+1}\right)}\delta^{\left(L_{i+1}\right)}$$

The third term in Equation (4) is problematic. It lies across the concatenation operation, which is nondifferentiable. As a result, the product of the first three terms, denoted as $\frac{\partial E}{\partial a^{\left(L_{i}\right)}}$ in Equation (6) below, cannot be computed directly by multiplication according to the chain rule.

(6) $$\frac{\partial E}{\partial a^{\left(L_{i}\right)}}=\frac{\partial E}{\partial y^{\left(L_{i+1}\right)}}\frac{\partial y^{\left(L_{i+1}\right)}}{\partial a^{\left(L_{ic}\right)}}\frac{\partial a^{\left(L_{ic}\right)}}{\partial a^{\left(L_{i}\right)}}$$

Although nondifferentiable, concatenation is an invertible operation. The proposed solution is to make use of gradient routing to unstack the concatenation, so as to link $\frac{\partial E}{\partial a^{\left(L_{ic}\right)}}$ in Equation (5) to $\frac{\partial E}{\partial a^{\left(L_{i}\right)}}$ in Equation (6). The transformation that gradient routing intends to achieve is shown in Equation (7) below.

(7) $$\frac{\partial E}{\partial a^{\left(L_{ic}\right)}}\to \frac{\partial E}{\partial a^{\left(L_{i}\right)}}$$

The very last term in Equation (4), i.e., $\frac{\partial a^{\left(L_{i}\right)}}{\partial y^{\left(L_{i}\right)}}$, is the derivative of the activation function. This derivative is known for activation functions that are common, such as sigmoid or ReLU [55]. It can be computed and then multiplied with the result of gradient routing in an element-wise manner. The result is the delta $\delta^{\left(L_{i}\right)}$ that was shown earlier on in Equation (4).

This completes the argument for backpropagation with gradient routing for the i-th layer. With delta $\delta^{\left(L_{i}\right)}$ now available, it can be used to compute the error gradient, which is then used to update the weights.

The gradient routing as aforementioned can be implemented by the proposed “split-slide-add” method. First, the error attributed to the concatenation sublayer is split into its preconcatenation parts. Then, the preconcatenation parts are aligned in time by sliding. Finally, the aligned parts are summed together.

**Figure 10:**
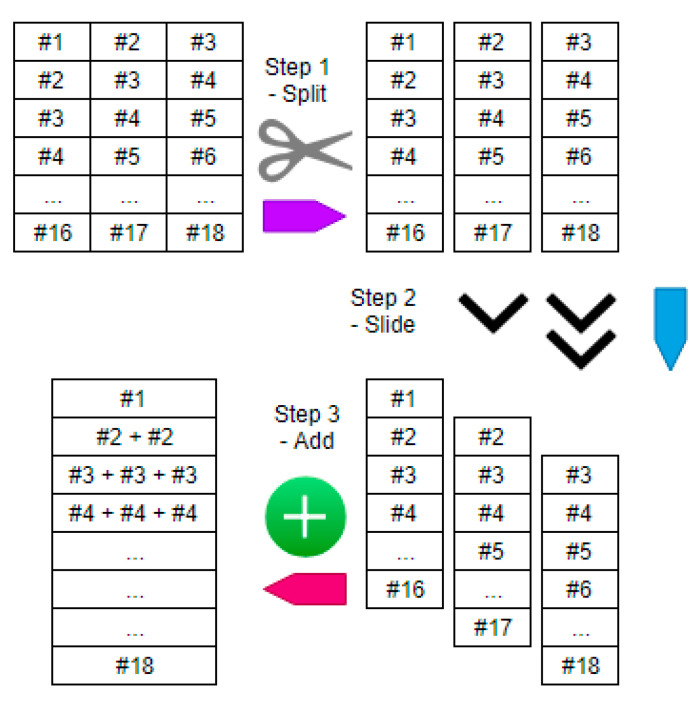
The three steps of the “split-slide-add” method for gradient routing.

To illustrate the “split-slide-add” method, consider the error contribution from the concatenation sublayer $L_{ic}$, i.e., $\frac{\partial E}{\partial a^{\left(L_{ic}\right)}}$ in Equation(5). Figure 10 below shows a table with 16 rows. Each of the rows in the table is the contribution of a particular concatenated vector in $L_{ic}$ to the error. There are 16 concatenated vectors in $L_{ic}$ in this example, as it is assumed here that the mini-batch size is 18 and that the concatenation is done with the time steps TS set to 3.

The first operation is to split the table into separate columns. Each of the columns are the contribution of the data before concatenation. The second operation, that of sliding the columns, aligns the preconcatenation parts according to their natural temporal order. This enables the summation in the third step to be meaningful. In summation, the values in the columns, now aligned in time, are added together. In consequence, $\frac{\partial E}{\partial a^{\left(L_{ic}\right)}}$ is transformed to $\frac{\partial E}{\partial a^{\left(L_{i}\right)}}$.

Gradient routing redistributes the error contribution from the concatenation sublayer $L_{ic}$ to the preconcatenation hidden layer $L_{i}$. It does not involve any learning of weight values. In other words, the amount of delta that the preconcatenation hidden layer receives is exactly the same as the delta passed to it from the concatenation sublayer. Thus, it will not cause overfitting in the proposed network.

With gradient routing done with the “split-slide-add” method, the proposed network will be able to learn about the temporal context that is passed through the network by the concatenation operation, even though it is a nondifferentiable operation.

The proposed methodology is different from the other convolutional neural networks used for time series classification. In those networks, the upsampling function used in the pooling layer for backward propagation of error is done based on the individual data instances. In contrast, in the deep temporal convolution network, the concatenation operation in the forward path, as well as the backpropagation with gradient routing in the backward path, are all done in mini-batches. These mini-batches keep the short-term temporal order of the data instances within them, and so the learning algorithm is able to learn the temporal context in them in the deeper layers of the network.

## 4. Data Experiments and Results

This section describes the data experiments that were done on two data sets from the UCI Machine Learning Repository [56], namely the EEG Eye State data set [57] and the human activity recognition (HAR) data set [1].

The data experiments on the EEG Eye State data set are presented here in three sections. Section 4.1 describes the spot-checking that was done to get the general benchmark of the data set and to verify the need to shuffle data; Section 4.2 shows the results of the 10-fold validation of the proposed network with TS values of 1, 2, and 5; Section 4.3 compares the performance of the proposed network with TS values of 2 and 5 with the DBN-DNN of equal complexity.

The data experiments on the HAR data set are arranged in two sections. Section 4.4 describes the general benchmark of the data set; Section 4.5 shows the results of the 10-fold validation of the proposed network with TS values of 2 and 5.

### 4.1. Spot Checking of the Eye State Data Set

The EEG Eye State data set is a multivariate numeric time series recorded from a single subject with a commercial EEG headset. It has 14 channels, corresponding to the fourteen electrodes of the EEG headset. After removing 4 outliers, the time series has 14,976 samples in it. Of these, 8254 samples (55.12%) correspond to the state of the eyes being open, and 6722 samples (44.88%) correspond to the state of the eyes being closed. The samples in the time series are related to each other in time, so the temporal context in the time series can be learnt by the proposed deep temporal convolution network.

The effect of shuffling and windowing are tested with 10-fold cross-validations with Python 3.6.5, Scikit-learn 0.19.1, and Keras 2.2.2. Four classification algorithms [58] in their default configurations are used, namely logistic regression (LR), k-nearest neighbor (KNN), decision tree (CART), and neural network (MLP).

Table 1 shows the results when there is no shuffling. Two sets of results are presented, one without windowing, and one with window length of 16 (125 millisecond) and a stride of 8.

As can be seen from Table 1, the accuracies are rather poor. Many of them are close to the random chance of 55.12% as might be produced by a zero-rule algorithm that always predicts the majority class. The high *p*-value of the Student’s paired t-test shows that there is no benefit in using the time delay representation when there is no shuffling.

The poor performance could be attributed to the classifiers learning the output patterns of the time series. To show the necessity of shuffling the data instances produced from a single time series, the same set of algorithms is run again, this time with the data instances in the data set randomized in order. The performance is shown in Table 2 below.

The accuracies are now significantly better. In particular, the MLP neural network in the default configuration (115 nodes in the hidden layer) achieved a classification accuracy of 95.2% without windowing and 97.4% with windowing. The low *p*-values shows that the time delay representation does improve the performance of the classifiers.

The results confirm the assertion in Section 2 that the sliding window method, used at the input to create the time delay representation, is sufficient for good performance in time series classification. The purpose of this work, however, is to go beyond the input layer to exploit the compositional locality of the time series data in the deeper layers of a network.

### 4.2. 10-Fold Validation of the Eye State Data Set with the Proposed Network, at TS = 1, 2, and 5

The proposed deep temporal convolution network (DTCN) is configured in this part of the work to have 224 nodes in the input layer, corresponding to a window length of 16 for each of the 14 electrodes, and just 20 nodes in the hidden layers, as shown in Table 3 below.

The small set of nodes in the hidden layers will have a negative effect on the performance, as less features will be extracted from the input by the network. However, in the proposed network, this will be counteracted by passing in the temporal context by concatenation to the deeper layers. As more discriminatory information will be available, the proposed network should be able to achieve better results, even though the number of nodes is made so few.

To confirm this, with the network configured as in Table 3 above, the DTCN is run with TS=1, 2, and 5. The first DTCN, with TS=1, has no concatenation and is equivalent to the DBN-DNN. Its performance serves as the benchmark of the other two DTCNs at TS=2 and 5.

The results for the DTCNs at TS=1, 2, and 5 are shown in Table 4 below. They are arranged in 10 folds so that the fluctuation across the folds can be seen.

From Table 4 above, it can be seen the performance of the DBN-DNN, i.e., DTCN at TS=1, is lower than the MLP shown in Table 2. This is not surprising, as the DBN-DNN is a very narrow network with only 20 nodes in the hidden layers. The performance of the DTCN gets better with the amount of concatenation increased to TS=2. When the amount of concatenation is increased further to TS=5, the improvement becomes obvious, as it is now better than the MLP. Not only is there an increase in the classification accuracies across the folds, the variance of the accuracies gets smaller also. This can be seen from the summary statistics of the 10-fold validation of the DTCNs at TS=1, 2, and 5, as shown in Table 5 below.

**Figure 11:**
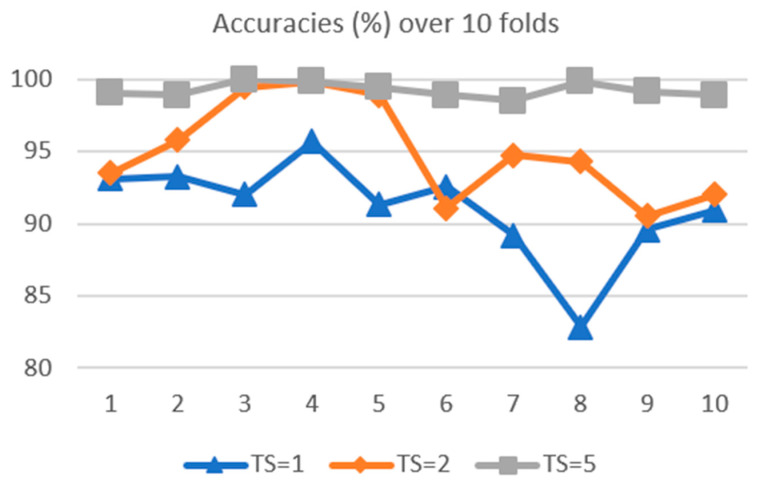
Classification accuracy over 10 folds, DTCN, *TS* = 1, 2 and 5.

Two observations can be made about the results when they are plotted in the line chart as shown in Figure 11 below: (1) the curve for TS=5 is higher, compared to the curves for TS=1 and TS=2, and (2) there is less fluctuation in the curve for TS=5.

The line chart in Figure 11 above shows that the bias is reduced (i.e., the classification has improved) and that the variance has reduced (less overfitting to the noise in the data). There is thus an improvement in the generalization performance [59] by the DTCN. This confirms the hypothesis that the concatenation of features at the deeper layers will provide the temporal context for better discrimination by the final classifier.

### 4.3. Comparing with Equivalent DBN-DNN

It is known that better performance can be achieved with a more complex network. A more complex network, such as a wider and deeper network, will also tend to extract more redundant information and so be more prone to overfitting [60]. To account for the improvement in performance due to higher network complexity, performance comparison should be made between networks of equal complexity.

In this part of the data experiment, comparison is made between DTCN at TS=2 and 5 and DBN-DNNs of equal complexity in terms of the number of network parameters [61]. The configurations of the equivalent DBN-DNNs are shown in Table 6 below. From the table, it can be seen that the number of hidden nodes has increased from 20 in Table 3 to 23 and 31 in Table 6.

The 10-fold validation results of the two equivalent DBN-DNNs are shown in Table 7 below. The summary statistics are in Table 8. Due to the higher network complexity, they have higher classification accuracy than the DTCN at TS=1 (refer to the first row of Table 4 and Table 5). This comes with an increase in variance, indicating some overfitting of the networks to the data.

**Figure 12:**
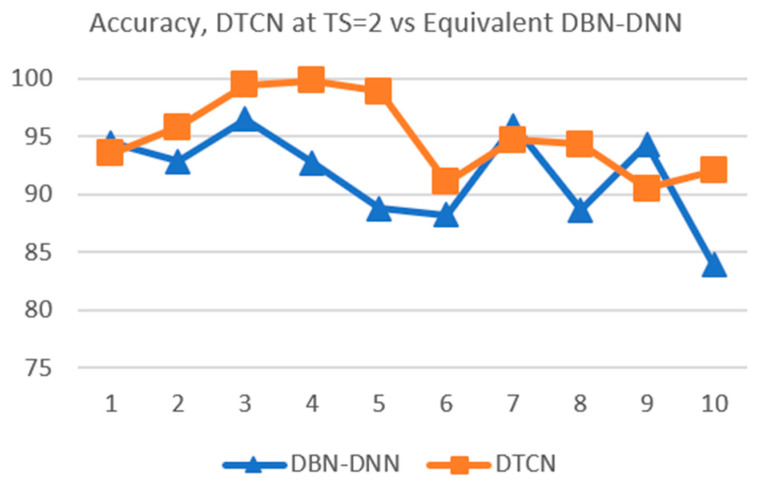
Performance of DTCN at TS=2 vs. equivalent DBN-DNN.

When the comparison is based on networks of the same complexity, it is found that the proposed deep temporal convolution network has better generalization performance than the equivalent DBN-DNNs. This can be seen from the line chart in Figure 12 for the DTCN at TS=2 and its equivalent DBN-DNN. The accuracy of the DTCN outperforms the equivalent DBN-DNN, and the variance of the DTCN is smaller than that of the equivalent DBN-DNN.

**Figure 13:**
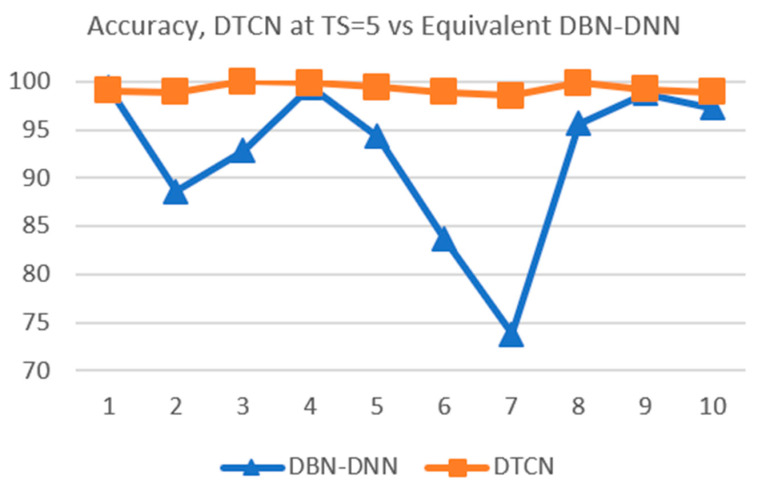
Performance of DTCN at *TS* = 5 vs. equivalent DBN-DNN.

The improvement in generalization performance is even more marked for the DTCN when the time steps is changed to TS=5, as shown by the line chart in Figure 13. This suggests that the amount of concatenation is the cause for the improvement.

By comparing the DTCN with the equivalent DNN-DBN, the confounding effect of network complexity can be discounted, and the benefit of passing in the temporal context to the deeper layers of the proposed network is confirmed.

### 4.4. Human Activity Recognition

This section describes the data experiment done on the human activity recognition (HAR) data set [1]. It is a motion sensor data set (accelerometer and gyroscope readings in three dimensions) based on the recordings of 30 subjects performing activities of daily living, as shown in Table 9 below.

In the data experiment, a fixed-width sliding window of 128 samples (2.56 s) and a slide of 64 samples (50% overlap) was used to create 10,299 data vectors from the data set. Each data vector has 768 samples in it, corresponding to 128 samples from each of the six readings (accelerometer and gyroscope in three dimensions).

To have a general benchmark of the data set, 10-fold cross-validation was done in Python with five different algorithms, namely (1) logistic regression, (2) k-nearest neighbor, (3) CART decision tree, (4) MLP neural network, and (5) ensemble by voting. All the algorithms were run in their default configurations in Python. In each fold, a random set of 3 subjects are used for testing and the other 27 subjects are used for training. The data were standardized in each of the folds during cross-validation. The data vectors in the data table are shuffled before they are used for training. The results are shown in Table 10 below.

There is certainly room for improvement in the performance. That could be done with the deep temporal convolution network (DTCN).

### 4.5. 10-Fold Validation of the HAR Data Set with the Proposed Network, at TS = 1, 2, and 5

In this data experiment, the DTCN is set at 768 nodes at the input layer, 200 nodes in the first hidden layer, 350 nodes in the second hidden layer, 200 nodes in the third hidden layer, and 12 nodes in the softmax layer, corresponding to the 12 activities to be classified.

Table 11 and Table 12 below show the classification accuracies of the deep temporal convolution network at TS= 1, 2, and 5.

It can be seen that the mean accuracy increased from 97.45 to 99.89% when concatenation is used in the DTCN by using a *TS* value of 2 instead of 1. This is accompanied by a corresponding decrease in the standard deviation from 0.48 to 0.05%. This shows that for the HAR data set, the proposed network does have better generalization performance when concatenation is introduced to the deeper layers of the network.

At TS=2, the DTCN is able to match the complex function of the HAR signal well and is able to learn the useful aspect of the highly varying data to achieve high classification accuracy. When *TS* is further increased to 5, overfitting [60] is likely to have occurred, which is seen in a drop in accuracy and an increase in variance, despite the increase in computational requirement. Due to the nonlearning nature of gradient routing and the weight-sharing nature of concatenation, it can be argued that overfitting, were it to occur, is due to the increased size of the neural network after concatenation is added to the network. This can be avoided by tuning the value of *TS* to within the range of 2 to 5.

## 5. Conclusions

The proposed network addresses the need in deep learning to match the data function of a time series data with an appropriate network structure. For nonstationary time series data such as physiological signals, the data function is highly varying, and so the composition of functions, as used in the proposed network, can be helpful in achieving better performance. To expose the temporal context and encourage the model to be shift-invariant, data processing is used in the proposed network, including 1. short term temporal order, 2. mini-batches that overlap, and 3. pooling of target labels through deeper layers. A matching learning algorithm by backpropagation with gradient routing is also proposed, with the “split-slide-add” operation being used for gradient routing.

The proposed network was tested with the electroencephalogram data set and the human activity recognition data set. The result shows that with the right amount of concatenation in the deeper layers of a network, it can generalize better than the equivalent DBN-DNN that uses just the time delay representation at the input layer. The proposed network is thus a useful way to classify time series data that originate from sensors, one that produces high accuracy without the need for manual feature crafting.

## Figures and Tables

**Table 1:** Mean classification accuracy and variance, without shuffling.

	Without Windowing	With Windowing	*p*-Value
LR	37.76% (19.38%)	36.44% (15.87%)	0.563
KNN	51.03% (13.33%)	53.81% (16.29%)	0.204
CART	50.67% (8.90%)	52.05% (8.48%)	0.516
MLP	50.38% (14.36%)	53.78% (17.48%)	0.453

**Table 2:** Mean classification accuracy and variance, with shuffling.

	Without Windowing	With Windowing	*p*-Value
LR	64.10% (0.771%)	62.20% (2.141%)	0.058
KNN	96.35% (0.413%)	95.80% (1.023%)	0.148
CART	83.93% (1.034%)	75.67% (2.597%)	0.000
MLP	95.20% (0.503%)	97.43% (1.117%)	0.001

**Table 3:** Configuration of deep temporal convolution network (DTCN).

	No. of Nodes
Input layer, $L_{0}$	224
First hidden layer, $L_{1}$	20
Second hidden layer, $L_{2}$	20
Third hidden layer, $L_{3}$	20
Softmax layer, $L_{c}$	2

**Table 4:** Cross-validation results (accuracies in %) of DTCN at TS = 1, 2, and 5.

Time Steps	Fold No.									
	1	2	3	4	5	6	7	8	9	10
TS=1	93.11	93.24	92.03	95.68	91.35	92.57	89.19	82.84	89.59	90.95
TS=2	93.51	95.81	99.46	99.86	98.92	91.08	94.73	94.32	90.54	92.03
TS=5	99.05	98.92	100.0	99.86	99.46	98.92	98.51	99.86	99.19	98.92

**Table 5:** Mean and standard deviations of 10-fold results of DTCN at TS= 1, 2, and 5.

Time Step	Mean	Std Dev
TS=1	91.05%	3.44%
TS=2	95.03%	3.42%
TS=5	99.27%	0.50%

**Table 6:** Configuration of Equivalent DBN-DNN for TS= 2 and 5.

	No. of Nodes in Equivalent DBN-DNN	
	TS=2	TS=5
Input layer	224	224
First hidden layer	23	31
Second hidden layer	23	31
Third hidden layer	20	20
Softmax layer	2	2
Total No. of Parameters	6181	8565

**Table 7:** Cross-validation results (accuracies in percentage) of equivalent DBN-DNN for TS= 2 and 5.

	Fold No.									
Eqv	1	2	3	4	5	6	7	8	9	10
TS=2	94.46	92.84	96.49	92.70	88.78	88.24	95.95	88.65	94.32	83.92
TS=5	99.32	88.65	92.84	99.46	94.32	83.65	73.78	95.68	98.78	97.30

**Table 8:** Means and standard deviations of 10-fold results of equivalent DBN-DNN for TS= 2 and 5.

	Mean	Std Dev
Eqv TS=2	91.64%	4.06%
Eqv TS=5	92.38%	8.26%

**Table 9:** Description of the 12 classes of activities in the human activity recognition (HAR) data set.

	Class	Description
Basic Activity	1	Walking
	2	Walking Upstairs
	3	Walking Downstairs
	4	Sitting
	5	Standing
	6	Laying
Transitional Activity	7	Stand to Sit
	8	Sit to Stand
	9	Sit to Lie
	10	Lie to Sit
	11	Stand to Lie
	12	Lie to Stand

**Table 10:** Classification accuracy and variance of the HAR data set, with shuffling.

Algorithm	Accuracy (in Percentage), 10-Fold Validated
logistic regression	64.822 (1.309)
k-nearest neighbor	77.009 (1.035)
CART	72.836 (1.465)
MLP	87.868 (0.870)
ensemble	85.29 (0.95)

**Table 11:** Cross-validation accuracies (%) of the HAR Data Set for DTCN at TS= 1, 2, and 5.

	Fold 1	Fold 2	Fold 3	Fold 4	Fold 5	Fold 6	Fold 7	Fold 8	Fold 9	Fold 10
TS=1	97.78	97.64	97.88	98.16	97.5	97.59	96.98	96.51	97.36	97.08
TS=2	99.83	99.96	99.91	99.91	99.96	99.83	99.91	99.83	99.87	99.87
TS=5	98.78	98.64	98.88	98.16	98.5	98.59	97.98	98.51	98.36	98.08

**Table 12:** Means and standard deviation of the cross-validation result of the HAR Data Set for DTCN at TS= 1, 2, and 5.

Time Step	Mean	Std Dev
*TS* = 1	97.45%	0.48%
*TS* = 2	99.89%	0.05%
*TS* = 5	98.45%	0.30%

## Data Availability

Publicly available datasets were analyzed in this study. The various datasets used are to be found in the cited references.
